# Physicochemical, Digestive, and Sensory Properties of Panax Notoginseng Saponins Encapsulated by Polymerized Whey Protein

**DOI:** 10.3390/foods10122942

**Published:** 2021-11-30

**Authors:** Zengjia Zhou, Xiaomeng Sun, Jianjun Cheng, Qingfeng Ban, Mingruo Guo

**Affiliations:** 1 Key Laboratory of Dairy Science, Northeast Agricultural University, Harbin 150030, China; m15255825668@163.com (Z.Z.); sunxm@neau.edu.cn (X.S.); jjcheng@neau.edu.cn (J.C.); qfban@neau.edu.cn (Q.B.); 2 Department of Nutrition and Food Sciences, College of Agriculture and Life Sciences, University of Vermont, Burlington, VT 05405, USA

**Keywords:** *Panax notoginseng* saponins, polymerized whey protein, nanoparticles, physicochemical properties, sensory properties

## Abstract

Panax Notoginseng Saponins (PNS) may be beneficial to human health due to their bioactive function. The application of PNS in functional foods was limited due to the bitter taste and low oral bioavailability. PNS were encapsulated by polymerized whey protein (PWP) nanoparticles. The physicochemical, digestive, and sensory properties of the nanoparticles were investigated. Results showed that the nanoparticles had a particle size of 55 nm, the zeta potential of −28 mV, and high PNS encapsulation efficiency (92.94%) when the mass ratio of PNS to PWP was 1:30. Differential Scanning Calorimetry (DSC) results revealed that PNS were successfully encapsulated by PWP. The mainly intermolecular forces between PNS and PWP were hydrogen bonding and electrostatic attraction confirmed by Fourier Transform Infrared Spectroscopy (FTIR). Results of simulated gastrointestinal digestion indicated that the PNS-PWP (1:30) nanoparticles had smaller average particle size (36 nm) after treatment with gastric fluids and increased particle size (75 nm) after treatment with intestinal fluids. Transmission Electron Microscopy (TEM) micrographs reflected that the nanoparticles had irregular spherical structures. The encapsulated PNS exhibited significantly (*p* < 0.05) decreased bitterness compared to the non-encapsulated PNS confirmed by the electronic tongue. The results indicated that encapsulation of PNS with PWP could facilitate their application in functional foods.

## 1. Introduction

The plant *Panax notoginseng* (Burk.) F.H. Chen is a genus of ginseng in the Araliaceae family. The components of *Panax notoginseng* include polysaccharides, nonprotein amino acids, flavonoids, and other components [1]. Panax notoginseng saponins (PNS) are considered the main biologically active components [2]. The effective activities of saponins result from the hydrolytic aglycone and secondary aglycone [3]. Studies have found that PNS have beneficial effects on curing cardiovascular diseases [4] and show anti-osteoporosis [5], anti-cancer [6], anti-inflammatory [7] activities as well as protecting action with regard to liver and kidney [8]. However, PNS are unacceptable to consumers due to the bitter taste, are unstable in the stomach, and present low oral bioavailability [9,10], which limits their applications in the food industry.

Encapsulation has been used to effectively mask the bitter taste of traditional Chinese medicines [11]. Encapsulation can protect biologically active components from degradation [12]. Nano-encapsulation systems have many advantages over traditional encapsulation systems, such as good solubility, high bioavailability, and controlled release of bioactive components during application [13,14]. Protein is an ideal wall material for its high nutritional value and safety [15]. It has been reported that the bitterness of Epigallocatechin-3-gallate (EGCG) was suppressed when encapsulated by β-lactoglobulin (β-Lg) [16]. Whey protein can be used as a suitable wall material to protect biologically active components due to its low price, resistance to gastric digestion [17,18], and ideal functional properties [19]. It was reported that whey protein-chitosan microcapsules were used to encapsulate squalene to improve the solubility and oxidation stability [20]. The pectin-whey protein complex has been used for the encapsulation of limonene [21]. PNS microspheres were prepared by a double emulsion method in a previous study [9]. However, the information about the utilization of PWP to encapsulate PNS hasn’t been reported.

The objectives of this study were to: (1) Prepare and characterize PNS-PWP nanoparticles using PWP as wall material; (2) Evaluate the stability of PNS-PWP nanoparticles under simulated gastrointestinal digestion; (3) Evaluate the bitterness of PNS and PNS-PWP nanoparticles by the electronic tongue.

## 2. Materials and Methods

### 2.1. Materials

Whey protein isolate (WPI) powder with a purity of 94% was purchased from Fonterra Co., Ltd. (Auckland, New Zealand). Panax Notoginseng Saponins (PNS, ≥80% purity) from the stems and leaves of *Panax notoginseng* were obtained from Xi’an Shengqing Biotechnology Co., Ltd. (Xi’an, China). The other chemicals and reagents used were all of the analytical grades.

### 2.2. Preparation of PNS-PWP Nanoparticles

PWP was prepared according to the method of Wang et al. [22], with some modifications. WPI powder was dissolved in deionized water to 10% (*w*/*v*) and kept stirring for 2 h. WPI solution was stored at 4 °C overnight to hydrate the protein. After recovering to room temperature, the WPI solution was adjusted to pH 7.0 by 1 M sodium hydroxide solution and heated at 80 °C for 30 min with stirring continuously to prepare the PWP solution. PWP solution was quickly cooled to 25 °C by an ice-water bath after heat treatment. Different amounts of PNS powder were added to PWP and stirred for 1 h to obtain PNS-PWP nanoparticles (the mass ratios of PNS to PWP were 1:50, 1:40, 1:30, 1:20, and 1:10, respectively). The prepared nanoparticles were determined directly or freeze-dried for the following tests. 

### 2.3. Determination of Encapsulation Efficiency

The encapsulation efficiency of PNS in PNS-PWP nanoparticles was determined according to the method of Wang et al. [23], with some modifications. The nanoparticles were dissolved in 10 mL methanol. 1 mL of solution was taken and centrifuged at 4000 rpm for 5 min at room temperature. After centrifugation, 100 μL of the supernatant were taken and blow dried by Nitrogen Blowing Apparatusat NDK-200-2N (Prosecution of Hangzhou Miou Instrument Co., Ltd., Hangzhou, China) at 40 °C for 10 min. 200 µL of 5% (*w*/*v*) vanillin-glacial acetic acid solution was used to dissolve the sample, and then 800 µL of perchloric acid solution was added. The sample was treated with nitrogen blowing at 60 °C for 15 min and immediately cooled down in an ice water bath. Finally, 5 mL of glacial acetic acid were added and mixed well. The absorbance of PNS at 551 nm was measured by a Dual-Beam UV-visible Spectrophotometer T9S (Beijing Puxi General Instrument Co., Ltd., Beijing, China) with methanol as blank. The PNS concentration was calculated according to the standard curve (*y* = 19.108*x* − 0.0923, *R*^2^ = 0.9986). The encapsulation efficiency is equal to the total PNS content minus the free PNS content divided by the total PNS content and multiplied by 100%.

### 2.4. Determination of Particle Size, Polydispersity Index (PDI), and Zeta Potential

All samples were diluted to 1 mg/mL with deionized water. The average particle size, PDI, and zeta potential were measured by a Malvern Zetasizer Nano ZS90 (Malvern Instruments Ltd., Worcestershire, UK) [24].

### 2.5. Fluorescence Spectroscopy

All samples were diluted to 0.2 mg/mL. The fluorescence spectrum was determined by the F-7100 Fluorescence Spectrophotometer (Hitachi Ltd., Tokyo, Japan). The excitation wavelength was set at 280 nm, and the emission wavelength at the range of 285 to 500 nm. The excitation slit and emission slit were 1 nm and 2.5 nm, respectively. 

### 2.6. Differential Scanning Calorimetry (DSC)

The thermal properties of PWP, PNS, and PNS-PWP nanoparticles were measured using a Differential Scanning Calorimetry instrument (DSC 3, Mettler Toledo, Switzerland). About 3 mg of sample were placed in an aluminum pan and set the starting temperature to 25 °C. The sample was heated from 25 °C to 300 °C at a rate of 25 °C/min and a nitrogen purge rate of 20 mL/min [25]. 

### 2.7. Fourier Transform Infrared (FTIR) Spectroscopy

The infrared spectra of PWP, PNS, and PNS-PWP nanoparticles were obtained by the Infrared Spectrometer (Nicolet iS10, Thermo Fisher, Waltham, MA, USA) using the potassium bromide (KBr) pellet method [26]. All samples were mixed with potassium bromide in a mass ratio of 1:100 and pressed into flakes. The spectrum at a wavenumber of 400–4000 cm^−1^, 64 scans, and a resolution of 4 cm^−1^ was obtained. The blank group was tested with KBr powder.

### 2.8. Stability of PNS-PWP Nanoparticles under Simulated Gastrointestinal Conditions

Simulate gastrointestinal digestion was operated according to the method of Yan et al. [27], with some modifications. The sample was mixed with hydrochloric acid (0.1 M) and stirred (100 rpm/min) at 37 °C for 10 min. The pH was adjusted to 1.5, and 10 mg of pepsin (3000 units/mg) were added to the sample. Samples were taken for analysis after 1 h of incubation. The pH was adjusted to 7.0, bile salt was added and shaken for 10 min, and then 20 mg of pancreatin (200 units/mg) were added to initiate the intestinal digestion. After 2 h, samples were collected for analysis. The average particle size, PDI, and zeta potential of the sample were measured.

### 2.9. Transmission Electron Microscopy (TEM)

The microstructure of the samples before and after digestion were observed by Transmission Electron Microscope H-7650 (Hitachi Ltd., Tokyo, Japan). The sample was diluted to 0.025 mg/mL with deionized water, then dropped onto a carbon-coated copper net, dyed, and freeze-dried for analysis. Images of the nanoparticles were captured at 100 kV with a magnification of 8000 [28].

### 2.10. Electronic Tongue Analysis

The Electronic Tongue SA402B (Insent Company, Atsugi-Shi, Japan) contains AAE (umami sensor), C00 (bitterness sensor), CA0 (sourness sensor), CT0 (saltiness sensor), and AE1 (astringency sensor) sensors, which were used to measure the taste attributes of the sample. The sensors were calibrated before the test. The equipment was washed with the cleaning solution for 90 s, and then washed twice with the reference solution for 120 s. The sensor was kept at the equilibrium position for 30 s. When the equilibrium condition was reached, 80 mL of sample were poured into the cup and tested for 30 s. The sensors were cleaned with a cleaning solution and reference solution after use. The sensor was inserted into a new reference liquid to test the aftertaste for 30 s.

### 2.11. Statistical Analysis

All experiments were performed three times. Comparisons among data of different groups were performed with one-way ANOVA. The results were presented as the mean ± standard deviation (SD). Duncan’s multiple range tests were applied to detect differences among the mean values of responses (*p* < 0.05).

## 3. Results and Discussion

### 3.1. Encapsulation Efficiency

**Figure 1:**
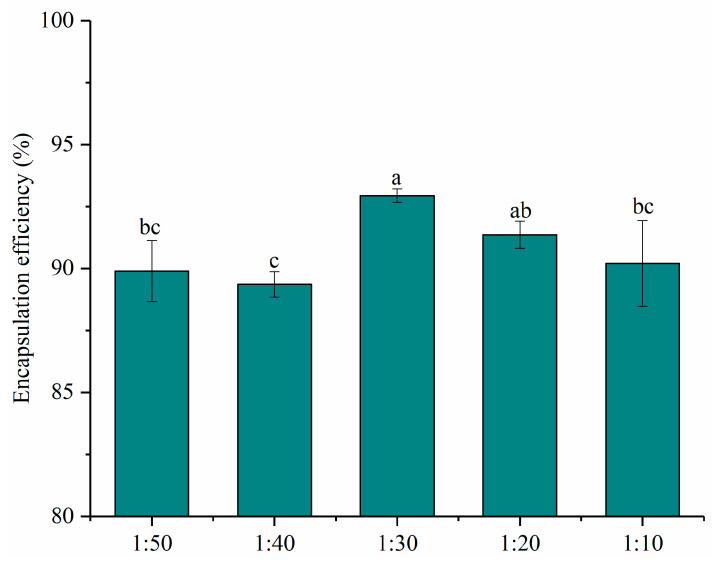
Encapsulation efficiency of nanoparticles at different PNS-PWP mass ratios of 1:50, 1:40, 1:30, 1:20 and 1:10 (*w*/*w*). Different subscript letters indicate a significant difference (*p* < 0.05).

The encapsulation efficiencies of nanoparticles with different mass ratios of PNS and PWP were given in Figure 1. All the encapsulation efficiencies of PNS were above 80%. The encapsulation efficiency of PNS increased significantly (*p* < 0.05) from 89.90% to 92.94% with PNS:PWP ranging from 1:50 to 1:30. However, the encapsulation efficiency of PNS decreased significantly (*p* < 0.05) from 92.94% to 90.2%, with the PNS:PWP ranging from 1:30 to 1:10. Results indicated that a part of the PNS was not encapsulated by PWP. Similar findings were reported by Tian et al. [29], who found that encapsulation efficiency of soy isoflavones (SIF) in polymerized goat milk whey protein (PGWP) nanoparticles decreased with increasing SIF concentration. Researchers found that the curcumin showed higher encapsulation efficiency (93.1%) and oxidation stability using whey protein as the wall material [30]. The PNS-PWP (1:30) nanoparticles were selected for subsequent study due to the highest encapsulation efficiency.

### 3.2. Changes in Particle Size and Zeta Potential 

**Figure 2:**
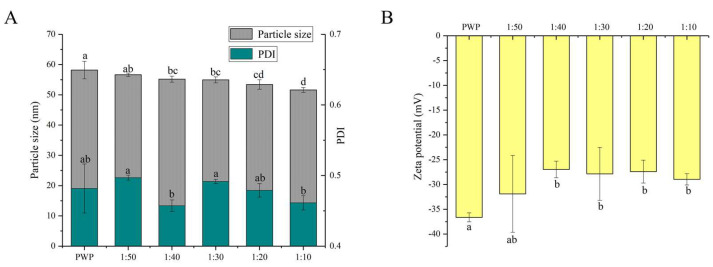
(**A**) Particle size, PDI and (**B**) zeta potential of PNS-PWP nanoparticles at different mass ratio of PNS to PWP. Different subscript letters indicate a significant difference (*p* < 0.05).

The functional properties of colloidal delivery systems are associated with the particle size, PDI, and charge characteristics [27]. It has been reported that nanoparticles with particle sizes in the range of 10–100 nm have unique advantages such as enhanced solubility and stability [31]. The average particle size and PDI of nanoparticles are shown in Figure 2A. 

All the samples showed an average particle size between 50 and 60 nm. A similar result was reported for the particle size of curcumin-loaded zein microparticles which presented less than 100 nm [28]. When PNS was added, the average particle size of PNS-PWP nanoparticles decreased compared to that of the PWP group. This observation can be explained by the fact that PNS and PWP were closely combined, forming a more compact structure. The result was consistent with that of Chen et al. [32], who reported that the particle size of quercetin encapsulated in zein nanoparticles was smaller than that of zein nanoparticles. The average particle size of nanoparticles decreased from 56 nm with PNS:PWP of 1:50 nanoparticles to 54 nm with PNS:PWP of 1:30 nanoparticles, but the difference was not significant (*p* > 0.05). When the mass ratio of PNS:PWP was 1:10, the average particle size of nanoparticles decreased significantly (*p* < 0.05) to 51 nm, compared that with the PNS:PWP of 1:30. This indicated that the average particle size of nanoparticles was related to the content of PNS.

The distribution width of particles was performed by PDI values. The PDI values of all samples were between 0.4 and 0.5, which indicated that the samples had good colloidal stability. Khan et al. [33] found that the PDI value of different content of 3,3’-diindolylmethane (DIM) in PWP nanoparticles was lower than 0.45, indicating that it has a narrow distribution and good stability.

The zeta potential value is an important parameter for system stability. As shown in Figure 2B, the zeta potential values of all the samples were negative. The zeta potential of PWP was around −36 mV. However, the zeta potential of all PNS-PWP nanoparticle increased to around −30 mV compared with that of the PWP group, which indicated that the system was in a stable state. There was no significant difference between nanoparticles with PNS:PWP of 1:50 and that with 1:10 (*p* > 0.05), which indicated that the large amount of PNS was encapsulated in PWP. Similar results were reported by Khan et al. [26]. 

### 3.3. Changes in Fluorescence 

**Figure 3:**
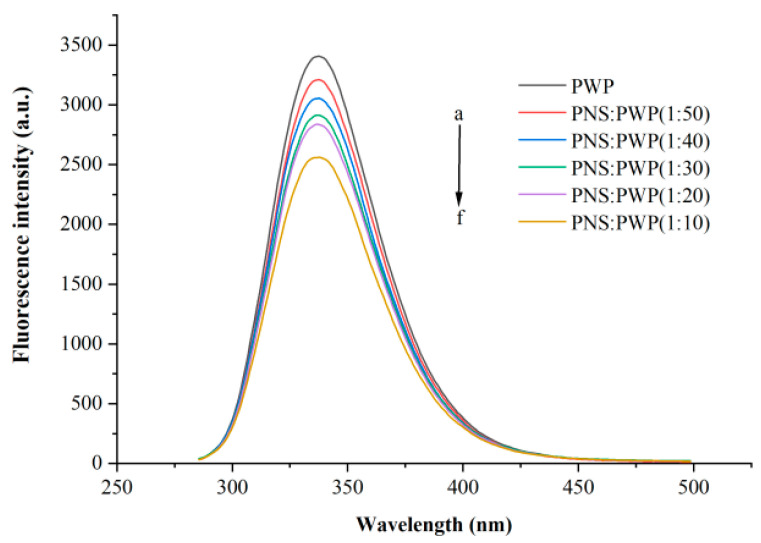
Intrinsic fluorescence spectra of PWP in the presence of different PNS mass. a–f: PWP, PNS:PWP (1:50), PNS:PWP (1:40), PNS:PWP (1:30), PNS:PWP (1:20) and PNS:PWP (1:10).

The interaction between small molecules and macromolecules can be studied by fluorescence quenching [34]. Fluorescence quenching can be segmented into static quenching and dynamic quenching [35]. Whey protein can emit fluorescence at a wavelength of 280 nm because it contains tyrosine and tryptophan [36]. Different amounts of PNS fluorescence quenching were investigated, as shown in Figure 3. It could be seen that PWP exhibited the highest fluorescence intensity. When the ratio PNS:PWP changed from 1:50 to 1:10, the fluorescence intensity decreased. This result can be attributed to PNS promoting fluorescence quenching of PWP, for example, rearrangement, energy transduction, and the formation of a ground-state complex [37]. Wu et al. [38] reported a similar phenomenon in that the fluorescence intensity decreased with increasing concentrations of Epigallocatechin (EGC).

### 3.4. Thermodynamic Properties of PNS-PWP by DSC

**Figure 4:**
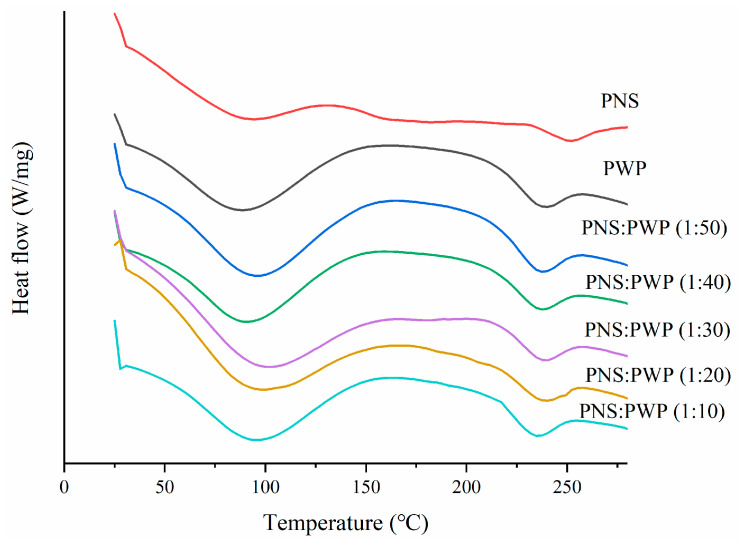
Differential scanning calorimeter (DSC) curves of PWP and PNS-PWP nanoparticles.

DSC curves of PNS, PWP, and PNS-PWP samples are shown in Figure 4. Results showed that PWP have an endothermic peak near 100 °C, which might result from the loss of water vapor. There was also an endothermic peak near 240 °C, which possibly resulted from the loss of chemically bond water [39]. PNS showed two endothermic peaks at 94.41 °C and 250.92 °C, respectively. The latter peak might be the melting peak of PNS [25]. Moreover, PNS-PWP nanoparticles with different mass ratios exhibited two endothermic peaks. The first peak temperature of the PNS-PWP samples with mass ratio from 1:50 to 1:10 was 94.35, 92.71, 100.14, 97.25, and 95.20 °C, respectively. The second peak temperature was 236.33, 236.81, 237.53, 238.34, and 233.86 °C, respectively. It could be found that the peak of PNS at about 250 °C disappeared in PNS-PWP nanoparticles, which might be because PNS were encapsulated into PWP nanoparticles. A similar result was reported by He et al. [40], who found that EGCG was successfully encapsulated in hordein nanoparticles. Liu et al. [41] also reported that curcumine was successfully encapsulated in zein-based nanoparticles. 

### 3.5. Spectrum of Fourier Transform Infrared Spectroscopy 

**Figure 5:**
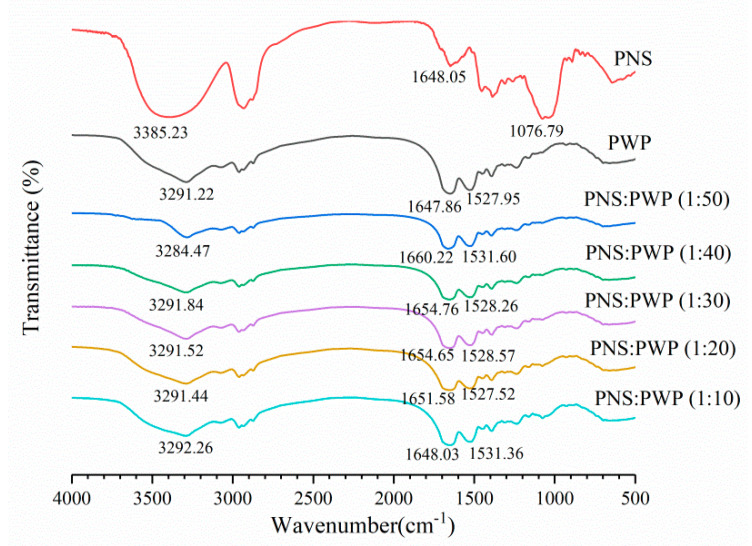
FTIR spectra of PWP, PNS and the mass ratio of PNS to PWP at 1:50, 1:40, 1:30, 1:20 and 1:10.

Infrared spectroscopy can be used to study the potential interaction between PWP and PNS. The infrared spectra of PWP, PNS, and PNS-PWP are shown in Figure 5. For whey protein, the band exceeding 3000–3600 cm^−1^ was related to intermolecular hydrogen bonding and O-H stretching vibration [42]. The range of amide I band and amide II band were 1580–1720 cm^−1^, and 1480–1580 cm^−1^, respectively [43]. The amide I band was mainly C=O stretch, and the amide II band was C-N stretch combined with N-H bending mode [44]. For the PWP sample, a characteristic peak was 3291.22 cm^−1^, this might correspond to O-H stretching vibration. For the PNS sample, one of the characteristic peaks appeared at 3385.23 cm^−1^. When PNS was added to PWP, the absorption peaks of PNS-PWP (1:50) nanoparticles to PNS-PWP (1:10) nanoparticles were shifted to 3284.47, 3291.84, 3291.52, 3291.44, and 3292.26 cm^−1^, respectively, which indicated that there may be a hydrogen bond generated between PWP and PNS. Moreover, the PWP sample had absorption peaks at 1647.86, and 1527.95 cm^−1^, which might belong to the amide I and amide II bands. The PNS sample showed absorption bands at 1648.05 cm^−1^. PNS caused the shift of amide I and amide II characteristic peaks of PNS-PWP nanoparticles, indicating that the presence of electrostatic interaction between PWP and PNS. Khan et al. [26] reported similar results that by comparing the spectra of WPI and encapsulated DIM, the amide I band shifted, indicating that there is an electrostatic attraction between WPI and DIM. The peak of the PNS sample at 1076.79 cm^−1^ disappeared in all PNS-PWP nanoparticles, which might be because PNS was encapsulated in PWP. Chen et al. [45] reported similar results that the peaks from 1000 to 1500 cm^−1^ of curcumin and quercetagetin scarcely appeared in the spectra of the Cur-zein-Que-HA (Curcumin-Zein-Quercetagetin-Hyaluronic acid) nanoparticles, which indicated that curcumin and quercetagetin were successfully encapsulated in the nanoparticles. Some of the characteristic peaks in the pure curcumin spectrum disappear when loaded with curcumin caseinate-coated zein (Cur-Z-C) nanoparticle, which might have been due to the fact that curcumin molecules were encapsulated in a protein matrix, rather than being surrounded by other curcumin molecules [27].

### 3.6. Stability of PNS-PWP Nanoparticles during Digestion In Vitro 

**Figure 6:**
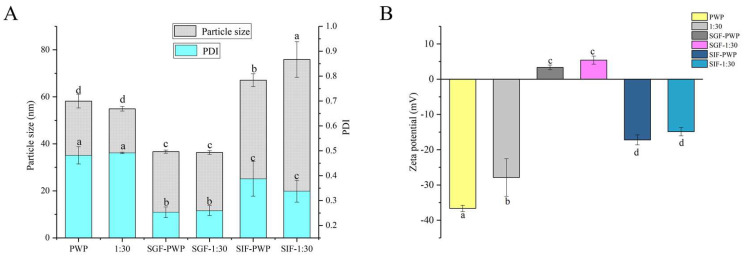
(**A**) Particle size, PDI and (**B**) zeta potential of PWP and PNS-PWP (1:30) before and after simulated gastrointestinal digestion. Different subscript letters indicate a significant difference (*p* < 0.05).

The simulated gastrointestinal stability of PNS-PWP nanoparticles is shown in Figure 6. The average particle size of PWP was significantly (*p* < 0.05) decreased from 58 nm to 36 nm after treatment with gastric fluids. The average particle size of PNS-PWP (1:30) nanoparticles also significantly (*p* < 0.05) decreased after digestion. This might have been caused by digestive enzymes and an acidic environment. However, there was no significant (*p* > 0.05) difference in particle size of the PWP and the PNS:PWP (1:30) nanoparticles in the gastric fluids. After digestion in simulated intestinal fluids (SIF), the average particle size of PWP and PNS:PWP (1:30) nanoparticles increased to 67 nm and 75 nm, respectively. This can be explained by the combined effect of digestive enzymes, pH environment, and bile salts that led to an increase in particle size [45,46]. The PDI values of all samples decreased after digestion. A similar phenomenon was discovered by Hu et al. [18]. Results suggested that the particle size distribution of nanoparticles became narrow, and PNS loaded nanoparticles were relatively stable.

In simulated gastric fluids (SGF), zeta potential is positive, which might be due to the low pH. In SIF, the zeta potential was negative. This indicated that the zeta potential of nanoparticles was affected by the microenvironment [18]. The zeta potential value of the PWP sample before and after the simulated gastrointestinal digestion was significantly (*p* < 0.05) changed. The absolute value of the zeta potential of PNS:PWP (1:30) nanoparticles was significantly (*p* < 0.05) decreased after simulated gastrointestinal digestion. However, the zeta potential values of PNS-PWP (1:30) nanoparticles had no significant (*p* > 0.05) difference in gastric fluids and intestinal fluids. 

### 3.7. Micrographs of Transmission Electron Microscopy (TEM)

**Figure 7:**
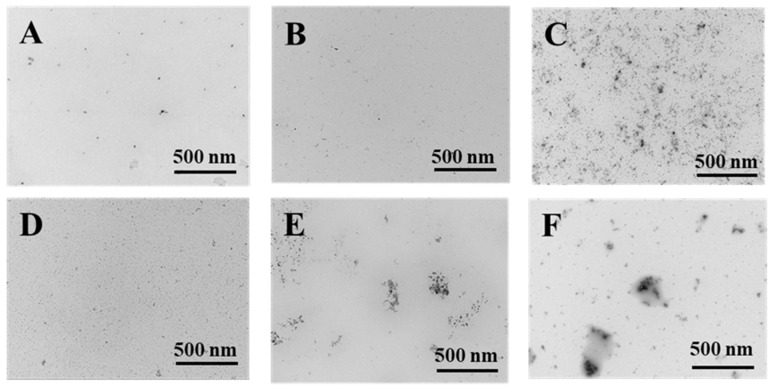
Transmission electron microscopy micrographs of PWP and PNS-PWP (1:30). PWP (**A**); PNS-PWP (1:30) (**B**); SGF-PWP (**C**); SGF-PNS:PWP (1:30) (**D**); SIF-PWP (**E**), and SIF-PNS:PWP(1:30) (**F**).

The TEM micrographs of PWP, PNS-PWP before and after digestion are shown in Figure 7. The morphology of all samples showed irregular spherical structure, which might be related to the pH of the solution and the isoelectric point of whey protein. The strands of aggregated proteins are favorably formed at pH deviating from the isoelectric point of proteins and low ionic strengths. [47]. At high concentrations, the initially formed strands of proteins might further aggregate to form larger macro-aggregates or fractal clusters [47]. The images showed that the average particle size of samples was in accordance with the results from dynamic light scattering (DLS). However, the average particle size was smaller than the results measured by DLS. The sample showed good dispersion before and after simulated gastric fluids digestion. After simulated intestinal fluids digestion, PNS-PWP (1:30) nanoparticles and PWP showed certain aggregation, which might be due to the effect of bile salt. In general, the digested samples were still stable.

### 3.8. Changes in Bitterness after Encapsulation

**Figure 8:**
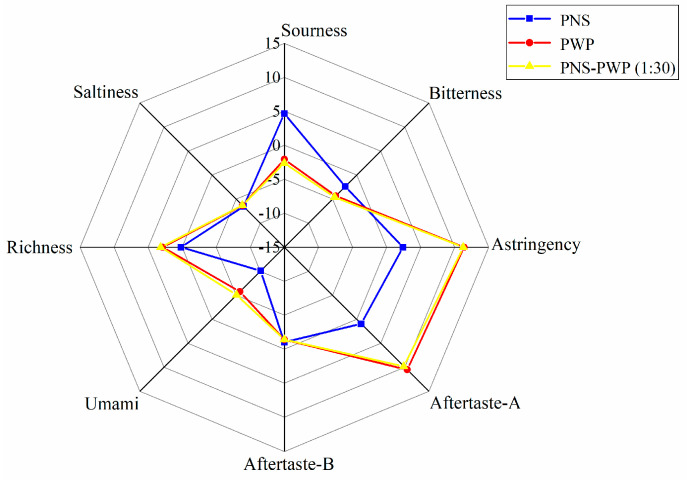
Radar chart of taste determination by the electronic tongue for PWP, PNS and PNS-PWP (1:30).

The response values of bitterness, saltiness, astringency, sourness, aftertaste-A, aftertaste-B, richness (umami aftertaste), and umami of PNS, PWP, and PNS-PWP (1:30) nanoparticles are shown in Figure 8. There was no significant difference in saltiness taste among the three groups (*p* > 0.05). The response values of astringency, richness, sourness, umami, aftertaste A and B were significantly different (*p* < 0.05) between PNS-PWP (1:30) nanoparticles and the non-encapsulated PNS group. The bitter value of the encapsulated PNS group was significantly decreased compared to the free PNS group (*p* < 0.05). A similar finding was reported by Shao et al. [48], who found the bitterness of caffeine was decreased after encapsulation by starch as wall material confirmed by the electronic tongue test. However, the response value of bitterness did not disappear completely, which might be due to the existence of some free PNS in the system. Moreover, there was no significant difference (*p* > 0.05) in the bitter value of PNS-PWP (1:30) nanoparticles compared with the PWP group, which indicated that PNS were encapsulated in PWP. Therefore, it is an effective carrier system to encapsulate PNS with PWP as wall material. 

## 4. Conclusions

The PNS-PWP nanoparticles were successfully developed, and the best mass ratio of PNS:PWP was 1:30 with the highest encapsulation efficiency. Interactions between PNS and PWP were hydrogen bonding and electrostatic attraction. The crystal melting peak of PNS disappeared in PNS-PWP nanoparticles, indicating that PNS was encapsulated in PWP nanoparticles. The nanoparticles were relatively stable under the simulated gastrointestinal digestion conditions. The bitterness of PNS has been effectively masked by PWP nanoparticles encapsulation. The prepared nanoparticles have the potential to be used in functional foods or used alone.
